# How Adolescent Sports Participation, Family, and School Resources Impact Later-Life Physical Activity Levels

**DOI:** 10.1093/geroni/igaa057.1626

**Published:** 2020-12-16

**Authors:** Thalida Arpawong, Margaret Gatz, Tara Gruenewald, Catalina Zavala, Dominika Seblova, Jennifer Manly, Susan Lapham, Carol Prescott

**Affiliations:** 1 University of Southern California, Los Angeles, California, United States; 2 Chapman University, Orange, California, United States; 3 Columbia University Medical Center, New York, New York, United States; 4 Columbia University, New York, New York, United States; 5 American Institutes for Research, Washington, District of Columbia, United States

## Abstract

Engaging in physical activity (PA) in adulthood has multiple protective health effects in later ages. However, unknown are the extent to which PA habits are laid down earlier in life and persist into adulthood, and the extent to which greater opportunities for PA during adolescence stem from differences in socioeconomic status (SES) which then affect opportunities for PA. We investigated potential mechanisms underlying these relationships using the longitudinal Project Talent Twin and Sibling Study (assessments in 1960 and 2014). With this design, we are able to hold genetics constant by modeling relationships between monozygotic twins (who share 100% of their genes) and therefore isolate effects of contextual factors on later-life PA (mean age ~70). We found that higher family SES (ß=.39, p<.0001) and sports participation in adolescence (ß=.05, p<.0001) predicted PA 55 years later, adjusted for gender and physical limitations. This held true when partialling out genetic variation that could otherwise explain these relationships. More education also predicted later-life PA (ß=.12, p<.0001) separately from family SES and partially mediated the effect of family SES on PA. While school-level resources (e.g., availability of sports and recreation opportunities) did not predict later life PA, they did associate with adolescent sports participation (ß=.26, p=0.007). Overall, later-life physical activity was influenced by earlier life sports participation and education, with family rearing resources being more important than high school resources. As twin pair correlations suggest gender differences, future research will examine whether family or school resources differentially benefit males or females for later-life physical activity.

